# Synergistic graphene-MnOx/honeycomb activated carbon (G-MnOx/HAC) and plasma technology for eradication of pathogenic microorganisms

**DOI:** 10.3389/fchem.2023.1207947

**Published:** 2023-08-02

**Authors:** Jiqing Zhang, Ying Jia, Xiaomeng Lv, Tiedan Xiong, Jun Su, Yuanzheng Huang, Keke Shen

**Affiliations:** ^1^ College of Missile Engineering, Rocket Force University of Engineering, Xi’an, China; ^2^ School of Marine Science and Technology, Harbin Institute of Technology, Weihai, China

**Keywords:** confined space, pathogenic microorganisms, G-MnOX-P/HAC, DBD plasma, eradication

## Abstract

This paper addresses the risk for environmental transmission of pathogenic microorganisms in confined spaces and the serious health hazards for personnel, and research on efficient eradication methods for the pathogenic microorganisms was carried out to provide technical support for ensuring the health of personnel in confined spaces. A series of graphene-MnO2 (G-MnO2) catalytic materials was prepared by hydrothermal and precipitation methods, and processing parameters such as the graphene doping method, the raw material ratio and the plasma action time were optimized. It was shown that G-MnOX-P/HAC prepared by a one-step precipitation method and with a graphene doping ratio of 10% had the best bactericidal effect in a dielectric barrier discharge (DBD) reactor after 4 min of reaction. The eradication rates for *Escherichia coli* (*E. coli*), *Staphylococcus aureus* (*S. aureus*), coronavirus and Aspergillus niger were all greater than 99.9%. The characterization techniques TEM, SEM, XRD, XPS, BET and FT-IR showed that the G-MnOX-P samples prepared by the one-step precipitation method had larger specific surface areas with more oxygen vacancies and functional groups on the surfaces, which was conducive to decomposition of the ozone generated by the dissociated plasma and formation of reactive oxygen species (ROS) for the microbial eradication process. Finally, by comparing the ozone-decomposition activity with the plasma co-catalytic performance, it was verified that efficient decomposition of the ozone facilitated the eradication of microorganisms. Based on this, an analysis of the mechanism for efficient eradication was carried out.

## 1 Introduction

Confined spaces are widely present in military installations and in all areas of everyday life. According to survey results, people spend more than 80% of their time in confined spaces, so the quality of the air in confined spaces is critical to human health (Yan and Lan, 2020; Lunati and Mucignat, 2022). Due to the restricted airflow between confined spaces and the outside world, liquids, vapours, gases, aerosols, solid particles and fumes can become harmful to the human body over time (Li et al., 2019). The toxic substances in the confined spaces mainly originate from materials stored in the confined space, including volatile toxic substances from welding, cutting, painting and other operations and living practices (Reponen et al., 1992; Van et al., 2018; Chillón et al., 2021). Airborne microorganisms include over a hundred species of bacteria, fungi and a small number of viral particles that of aerosols, and 50.5% of bacteria and 49.5% of fungi are present as bioaerosols in confined spaces. Fungi are the main air polluters of indoor air, including Aspergillus, Penicillium, Aspergillus spp. and yeasts, and the bacteria are predominantly Gram-positive bacteria (Chen et al., 2020) [7]. Eradication of the pathogenic microorganisms in confined spaces is an important part of microenvironmental air quality control and is of far-reaching significance for national defence construction (National Research Council, 2005; William, 2014; Dbouk and Drikakis, 2021). Microbial pollution prevention and control measures for confined spaces include ventilation, filtration and sterilization manipulations, ultraviolet irradiation, plasma generation, photocatalytic sterilization, and disinfection with ozone, peroxyacetic acid, or chlorine dioxide (Brągoszewska et al., 2020; Song et al., 2022).

Low-temperature plasma sterilization is a novel sterilization technology based on the characteristics of the low-temperature plasma itself, such as low temperature application and sterilization efficiency, and it has been applied in many areas, such as agricultural product processing and sterilization within food packaging (Ehlbeck et al., 2011; Machala et al., 2012; Ben et al., 2017). The plasma contains many electrons and charged groups, as well as charged ions. The electrons and charged ions produced in the plasma have high energies, and attack by these charged species on the outer cell membrane can cause breakdown etching of the outer cell wall of the bacterium and eventually destroy the tensile strength of the outer cell membrane, leading to cell rupture (Sun et al., 2018; Wang et al., 2022).

The electrons generated within the DBD field ionize the air, and the bonds of molecular oxygen break to form individual oxygen atoms, which then combine with molecular oxygen to produce ozone (Loiseau et al., 1994; Park et al., 2016). Eqs. 1–3 demonstrate the reaction process, where M represents any of the particles in the discharge region. However, when suitable catalytic conditions are established, ozone can be decomposed on the catalyst surface via a two-step reaction, this is shown in Eqs. 4, 5, where O* and O2* are ROS on the catalyst surface (Aziz et al., 2018; Hajer et al., 2022; Cheng et al., 2023). The reaction forms ROS with strong oxidation capacity, and ROS has a significant effect on microbial eradication as well as on the treatment of pollutants (Yuan et al., 2018). In addition, the ozone produced in plasmas containing oxygen sources has a relatively long lifetime (Khuntia et al., 2021), and DBD plasma is among the most effective methods for generating ozone (Kamel et al., 2018; Xia et al., 2022). Manganese oxides exhibit good catalytic activity in ozone oxidation, and the specific surface area of the catalyst and the valence distribution of the elements on the surface are important factors affecting the catalytic activity, which can be improved with better preparation processes or doping (Gong et al., 2017; Jia et al., 2023).

$$e^{-}+O_{2}\to O+O+e^{-}$$ (1)

$$e^{-}+O_{3}\to O+O_{2}+e^{-}$$ (2)

$$O+O_{2}+M\to O_{3}+M$$ (3)

$$O_{3}\to O_{2}+O^{*}$$ (4)

$$O_{3}+O^{*}\to O_{2}+O_{2}^{*}$$ (5)

In this study, a combination of DBD and a highly efficient manganese-based catalyst was used. G-MnO_x_/HAC technology was developed for the eradication of pathogenic microorganisms in a plasma field. First, a MnO_x_/HAC catalyst was obtained via different preparation processes using HAC as the catalyst carrier and MnO_x_ as the active component, the efficacy of *Escherichia coli* eradication in plasma is investigated. The physical and chemical properties, such as the crystalline structure, surface element distribution and specific surface area, of the catalyst were characterized to determine the effects of the preparation process on the catalytic activity. To achieve more efficient oxidative eradication of pathogenic microorganisms, the manganese-based catalysts were doped and modified with graphene to elucidate the effects of surface functional groups, specific surface area and valence distribution of the surface elements on the catalytic activity. Based on this, the G-MnO_x_/HAC materials were tested for performance and reproducibility against different microorganisms, and a new method was developed for synergistic eradication of microorganisms in plasma fields.

## 2 Materials and methods

### 2.1 Materials

Manganese sulfate (MnSO_4_) and honeycomb activated carbon (HAC) were purchased from Shanghai Titan Scientific Co., Ltd. The alumina sol was purchased from Zhitai Nano Micro New Material Co., Ltd. The graphene was made in the laboratory. Potassium permanganate (KMnO_4_) was purchased from the Harbin City Xinchun Chemical Plant. Deionized (DI) water was made in the laboratory. All chemicals were of analytical grade and were used without further purification.

### 2.2 Preparation

#### 2.2.1 Preparation of MnO_2_ by the hydrothermal method (MnO_2-H_)

KMnO_4_ (0.012 mol) and MnSO_4_ (0.00625 mol) were dissolved in 100 mL of water and stirred for 40 min with a magnetic stirrer to mix them thoroughly. Then, the mixed solution was transferred to a reaction kettle and heated in an oven at 140°C for 12 h. After cooling to room temperature, the mixture was removed, vacuum filtered, washed with DI water to neutrality, and finally dried at 60°C. MnSO_4_ was added to the aluminium sol and then loaded onto the HAC and dried overnight.

#### 2.2.2 Preparation of MnO_2_ by one-step precipitation (MnO_2-P_)

KMnO_4_ (0.0185 mol) was dissolved in 50 mL of DI water and 0.026 mol of MnSO_4_ was dissolved in 15 mL of water. The KMnO_4_ solution was slowly added dropwise to the manganese sulfate solution and stirred for 40 min with a magnetic stirrer to mix it thoroughly. After the reaction, the solution was vacuum filtered, washed with DI water to neutral, and finally dried at 60°C. Some of the MnO_2-P_ was mixed with the aluminium sol and then loaded onto the HAC and dried overnight.

#### 2.2.3 Preparation of graphene-modified graphene-MnO_X-P_/HAC by a one-step precipitation method (G-MnO_X-P/_HAC)

KMnO_4_ (0.0185 mol) was dissolved in 50 mL of DI water, and 0.026 mol of MnSO_4_ and a certain proportion of graphene powder were dissolved in 15 mL of water, and the KMnO_4_ solution was slowly added in dropwise fashion to the MnSO_4_ solution and stirred for 40 min with a magnetic stirrer. At the end of the reaction, the product was filtered under vacuum, washed with DI water to neutral, and finally dried at 60°C. A certain amount of MnO_2-P_ was then mixed with the aluminium sol, loaded onto the HAC and dried overnight to obtain G-MnO_X-P/_HAC.

#### 2.2.4 Preparation of graphene-modified graphene-MnO_X-M_/HAC by mixing (G-MnO_X_-_M_/HAC)

The G-MnO_2-P_ prepared by the one-step precipitation method was mixed with graphene powder at a certain ratio to obtain G-MnO_X-M_ samples with different graphene doping ratios. Then, a certain amount of G-MnO_X-M_ was mixed with the aluminium sol, loaded onto the honeycomb activated carbon and dried overnight to obtain G-MnO_X-M_/HAC.

### 2.3 Characterization methods

X-ray diffraction (XRD) studies were carried out with a Nissan Rigaku D/MAX RA X-ray polycrystalline diffractometer under the following conditions: the source was a copper target (Cu Kα, *λ* = 0.15418 nm), and the scan range was 10°–80°. The specific surface areas and pore structures were determined with the JW-BK132F Beijing Jingwei Gao Bo Automatic Static Volume Method for Specific Surface and Pore Size Analyses with N_2_ adsorption at 77 K. The specific surface area, pore volume and pore size distributions of the catalyst were obtained with the Brunauer–Emmett–Teller (BET) and BJH models, respectively. Scanning electron microscopy (SEM) with a Phillips XL-30-ESEM scanning electron microscope was used to observe the catalysts. Transmission electron microscopy (TEM) was performed with a JEM-2010 system from JEOL, Japan, with an accelerating voltage of 160 kV. Fourier transform infrared spectroscopy (FT-IR) was carried out with a Bruker Tensor 27 r. The spectral range was 4000–600 cm^-1^, and 32 scans were collected. The catalyst was mixed with KBr and pressed into tablets for the FT-IR analyses. X-ray photoelectron spectra (XPS) were measured with a Thermo ESCALAB 250 photoelectron spectrometer using a monochromatic Al Kα irradiation source (hv = 1,486.6 eV), a power of 150 W, a 500 μm beam spot, and an energy analyser with a fixed transmission energy of 30 eV.

### 2.4 Eradication experiments

#### 2.4.1 *Escherichia coli*/*Staphylococcus aureus/Aspergillus* niger eradication experiment

A certain amount of material was placed in a small sterile dish measuring 3.5 cm in diameter and spread out, and 3.0 mL of phosphate buffered saline (PBS) followed by 0.1 mL of bacterial or viral stock solution were injected. After 2–10 min of interaction, the liquid in the Petri dish was transferred to a 5.0 mL test tube. A total of 0.1 mL was aspirated directly into the dish, and the plate count method was used to determine the number of viable bacteria. Another dish was taken and lined with a blank carrier and filled with 3.0 mL of PBS followed by 0.1 mL of bacterial fluid for use as a positive control group. The subsequent testing and live culture count procedures were the same as those described above for the test group.

The tests were repeated 3 times, including those for the control, and the number of viable bacteria and the eradication rate were calculated for each group.

#### 2.4.2 Coronavirus eradication experiment

The method was the same as 2.4.1, and the counts were performed using the tissue culture infective dose (TCID50) method for virus detection.

#### 2.4.3 Environmental silo eradication experiment

Eradication experiments with the pathogenic microorganisms were performed in an environmental compartment with reference to Section 2.1.3, Air disinfection effect identification experiment, in the Disinfection Technical Code of the People’s Republic of China, 2002 edition. The parameters for this experiment included a volume of 1 m^3^, an action time of 1 h, 200 g of material, an ambient temperature of 20°C–25°C and a humidity of 50%–70%. The eradication rate was calculated as follows:

$$N_{t}=\frac{V_{0}-V_{t}}{V_{0}}\times 100\%$$ (6)

$$K_{t}=\frac{V_{0}^{\prime }\left(1-N_{t}\right)-V_{t}^{\prime }}{V_{0}^{\prime }\left(1-N_{t}\right)}\times 100\%$$ (7)

where: N_t_ is the natural extinction rate of airborne bacteria, V_0_ and Vt and the airborne bacterial contents at different times before and during the control test, respectively, K_t_ is the eradication rate of airborne bacteria with the disinfection treatment, and V_0_’ and V_t_’ are the airborne bacterial contents before and during the disinfection process of the test group, respectively.

## 3 Results and discussion

### 3.1 Screening of the preparation methods

By examining eradication of the pathogenic microorganisms with separate plasmas and plasmas together with MnO_2_ prepared via different methods in Table 1, it was found that the added catalyst greatly improved the eradication rate from 94.10% without a catalyst to over 99%. The MnO_2-P_ sample showed the best effect with an eradication rate of 99.90%, indicating that this sample had an excellent synergistic effect. The main sources of the excellent performance and the mechanism of influence are elucidated below through characterization of the catalysts.

**TABLE 1:** *Escherichia coli* eradication rate of manganese oxide with different preparation methods.

Materials	Blank group (%)	MnO2-H (%)	MnO2-P (%)
Eradication rate	94.10	99.10	99.90

**FIGURE 1:**
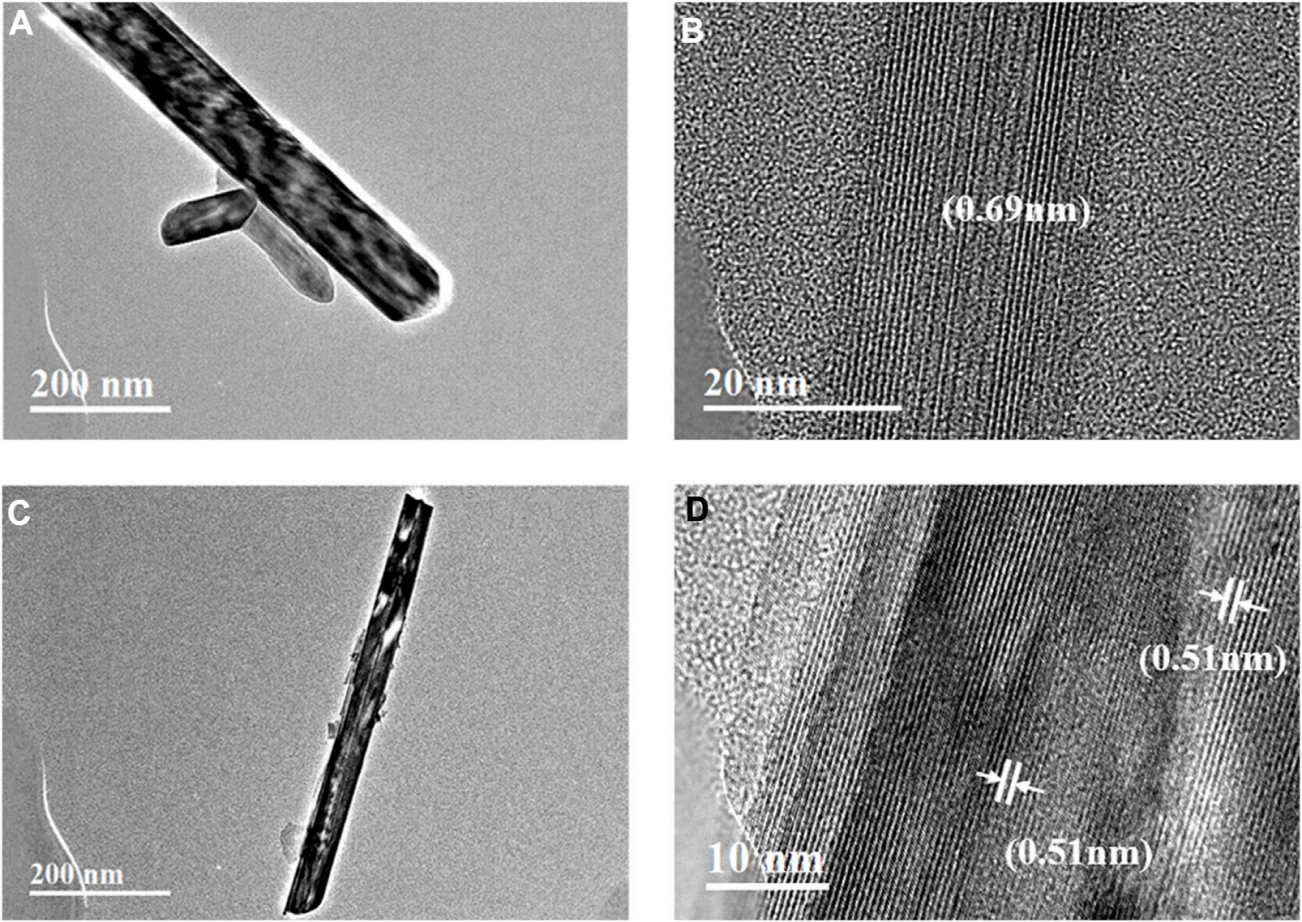
TEM image of **(A, B)** MnO_2-H_ and **(C, D)** MnO_2-P_.

The microscopic morphologies of the MnO_2_ samples obtained by the two different preparation methods were characterized with TEM, and MnO_2-H_ (Figure 1A, B) showed elongated nanowires with lengths of approximately 0.85 μm, diameters of approximately 50–80 nm, grain surface stripe spacings of 0.69 nm and an exposed (110) surface, compared to the aforementioned samples, the MnO_2-P_ (Figure 1C, D) samples had rougher surfaces with more defects, shorter nanowire lengths of approximately 0.61 μm, grain surface stripe spacings of 0.51 nm and an exposed (001) surface. MnO_2-P_ had a rougher surface and more defects than the other samples, shorter nanowire lengths of approximately 0.61 μm, 0.51 nm streak spacings and a (001) exposed crystal surface. In summary, the hydrothermal MnO_2-H_ catalyst exhibited longer particles, relatively larger particle sizes and an overall nanowire shape, while MnO_2-P_ had a rougher surface with more defects. This facilitated the formation of a larger specific surface area, which was conducive to the decomposition of ozone on the catalyst surface, this facilitated ROS participation in the microbial eradication process, resulting in a higher eradication effect for MnO_2-P_.

**FIGURE 2:**
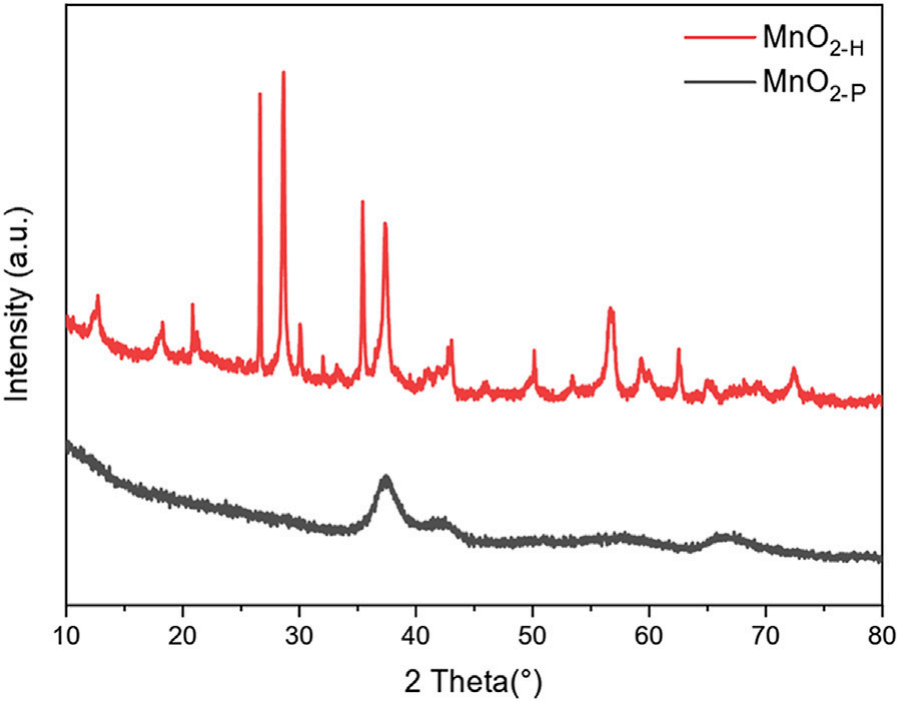
XRD pattern of the sample.

Figure 2 shows the XRD patterns for MnO_2_ samples prepared with the two different preparation processes. By comparing the spectral data of these two samples with the data on the standard card, it was found that the lattice parameters for MnO_2-H_ corresponded exactly to those for α-MnO_2_ (JCPDS 44–0,141), with distinct diffraction peaks at 12.7°, 28.7° and 36.7° corresponding to the (110) (310) and (211) crystal planes, respectively. The diffraction peaks for MnO_2-P_ were relatively broad and indicated poor crystallinity, and the sample was identified as α-MnO_2_ (JCPDS 44–0,141) by comparison with the standard card, the main diffraction peak was at 36.7° and corresponded to the (211) crystal plane. In summary, based on the above analyses, the samples obtained from the two different preparation processes used in this study were all MnO_2_. The hydrothermal method produced a more fully grown and impurity-free sample with higher crystallinity and distinct diffraction peaks. By comparing the MnO_2_ samples prepared by the hydrothermal method with those prepared by the one-step precipitation method, it was found that the crystallinity of MnO_2-P_ was lower, indicating that there may be many defects and unsaturated interfaces on its surface. These may be catalytically active sites for decomposition of the ozone, leading to significant increases in the catalytic activity.

**FIGURE 3:**
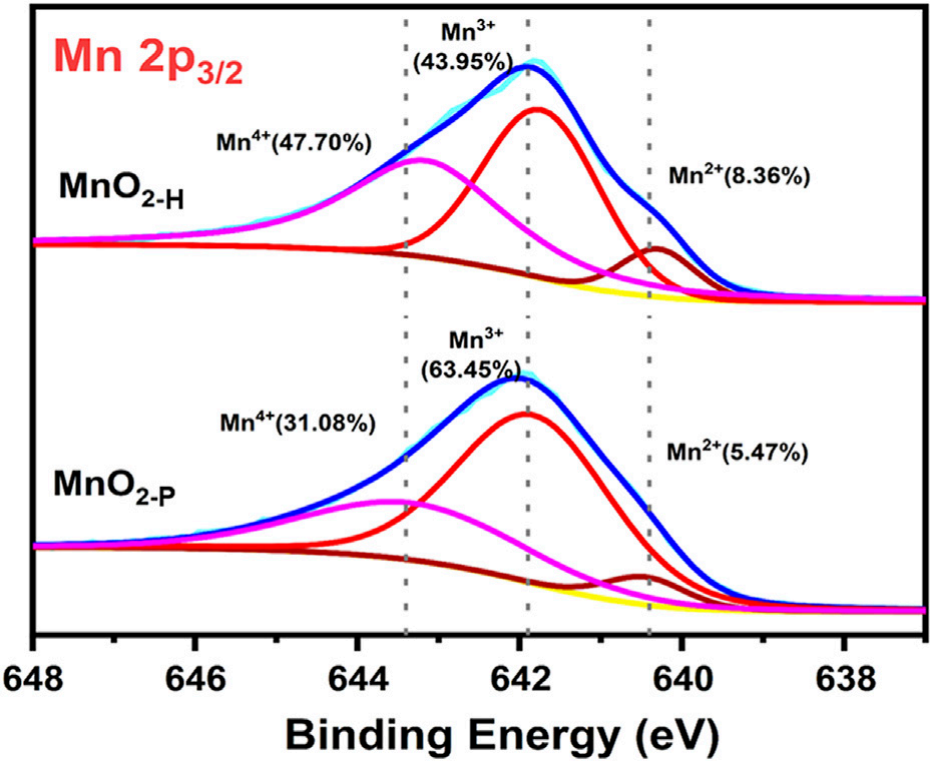
Valence distribution of Mn elements in sample.

Figure 3 shows that the peaks were not symmetrically distributed, indicating that there were multiple valence states in the samples. The common valence states for manganese in the manganese oxides are Mn^2+^, Mn^3+^ and Mn^4+^, which were identified with the binding energies indicated for Mn^2+^ (640.2 eV), Mn^3+^ (641.8 eV) and Mn^4+^ (643.2 eV) after fitting the results. The valence distributions of the different crystalline samples were significantly different, the relative proportions of the three ion contents were calculated from the peak areas, and the results are shown in Figure 3. The MnO_2-P_ sample exhibited the highest proportion of lower valent Mn species and a (Mn^2+^+Mn^3+^)/Mn^4+^ ratio of 2.21, which was much higher than that of MnO_2-H_. In general, the presence of low valent manganese species (Mn^2+^ or Mn^3+^) in MnO_2_ results in the formation of oxygen vacancies on the surface of the sample in order to maintain charge neutrality, and this can be used to assess the relative amount of oxygen vacancies. It is clear that the one-step precipitation method created more surface oxygen vacancies, which was consistent with the lower crystallinity indicated by the XRD data.

**FIGURE 4:**
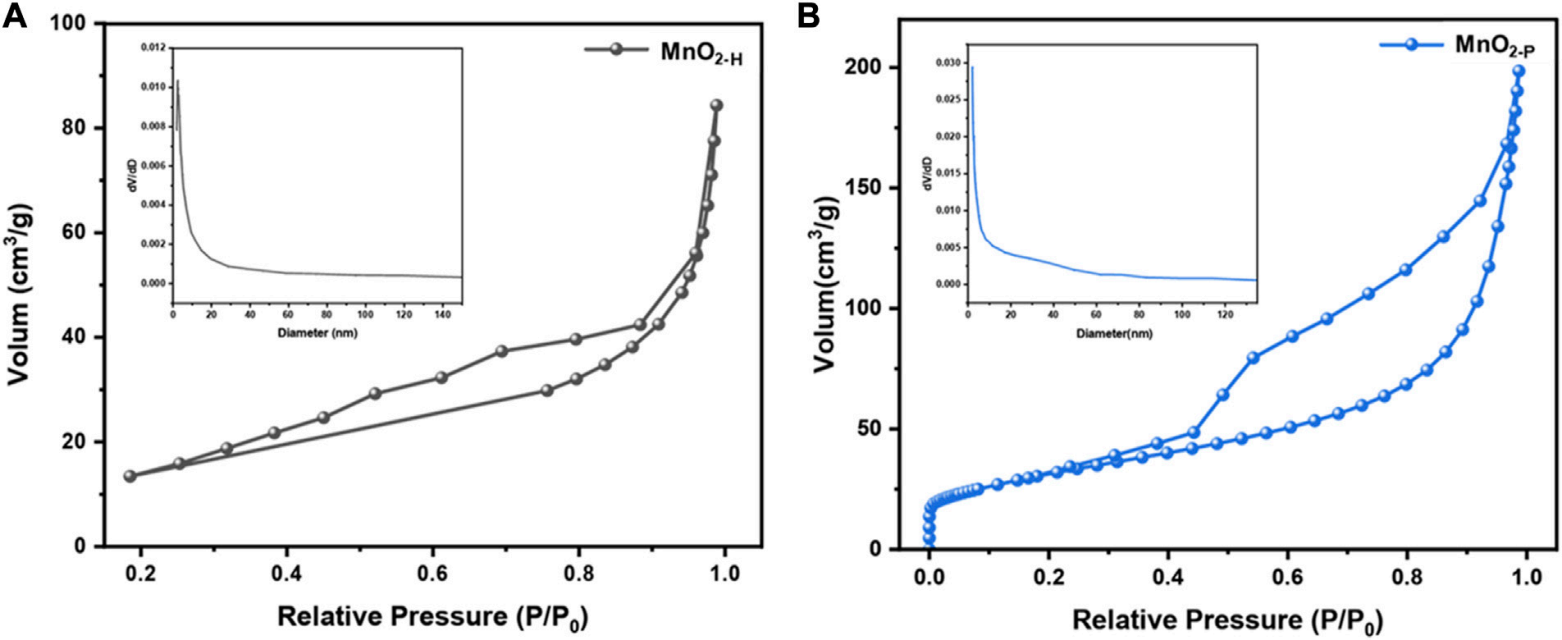
**(A)** MnO_2-H_ and **(B)** MnO_2-P_ adsorption and desorption curves (inset shows pore size distribution).

The catalytic ozone decomposition reaction is a heterogeneous catalytic process in which the adsorption properties of the catalyst strongly influence the catalytic activity (Li et al., 2020). The first process to occur in this sequence is adsorption of ozone onto the catalyst surface and dissociation to form oxygen molecules and O*, followed by interactions of the adsorbed species O* with ozone, and then desorption of the final product from the catalyst surface to complete the ozone decomposition process. The structural parameters of the catalyst, such as the specific surface area and pore structure, affect the overall adsorption performance. A N_2_ adsorption/desorption analysis was used to determine the specific surface areas and pore sizes of the catalysts. As shown in Figure 4, both samples exhibited H_3_ hysteretic ring IV adsorption isotherm curves, indicating that both catalysts were mesoporous materials. Table 2 shows the specific surface areas and ore volume of the samples. MnO_2-P_ showed a higher specific surface area (113.25 m^2^/g), which was approximately 1.3 times that of MnO_2-H_ (49.29 m^2^/g) prepared by the hydrothermal method, and the specific surface areas of the samples were consistent with the microbial eradication activities. It is generally accepted that a larger specific surface area favours ozone adsorption on the catalyst and facilitates the catalytic process, which is in good agreement with the results of the experiment.

**TABLE 2:** The specific surface areas and pore volume of the samples.

Catalyst	Specific surface areas (m2/g)	Pore volume (cm3/g)
MnO2-H	49.29	0.13
MnO2-P	113.25	0.31
G-MnO2-M	121.17	0.33
G-MnO2-P	138.06	0.37

The analysis showed that G-MnO_2-P_ had better microbial eradication properties because of its larger specific surface area and larger pore volumes and sizes, as shown in Table 2.

### 3.2 Parameter optimization for the G-MnO_X_/HAC materials

The experiments used to determine the effects of the different graphene doping methods on the eradication rates for *E. coli* were designed to test the different doping methods for MnO_2_, as shown in Table 3. The G/MnO_2-P_ produced by the one-step precipitation method had the best *E. coli* eradication performance, with a 99.93% eradication rate. The mechanisms and factors leading to the excellent performance were determined by characterization of the catalysts, as described below.

**TABLE 3:** Effect of different doping methods of graphene on the eradication rate of *Escherichia coli*.

Materials	MnO2-P (%)	G-MnO2-P (%)	G-MnO2-M (%)
Eradication rate	99.90	99.93	99.90

**FIGURE 5:**
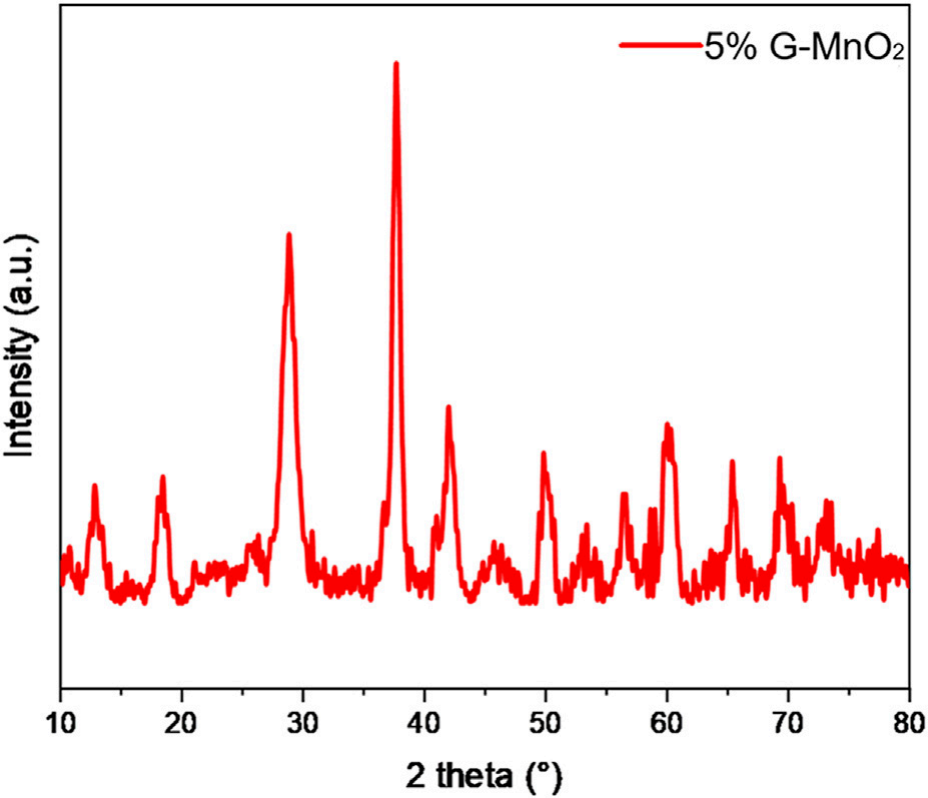
XRD spectrum of 5% G-MnO_2_ sample.

Figure 5 shows the XRD patterns for the MnO_2-P_ samples. A comparison with the XRD standard card for α-MnO_2_ (JCPDS PDF44-0,141) showed that the synthesized samples were all α-MnO_2_ crystalline phases with the tetragonal crystal system. In addition, no diffraction peaks for graphene were present, probably due to the low doping level and the uniform dispersion of graphene on the surfaces of the samples.

**FIGURE 6:**
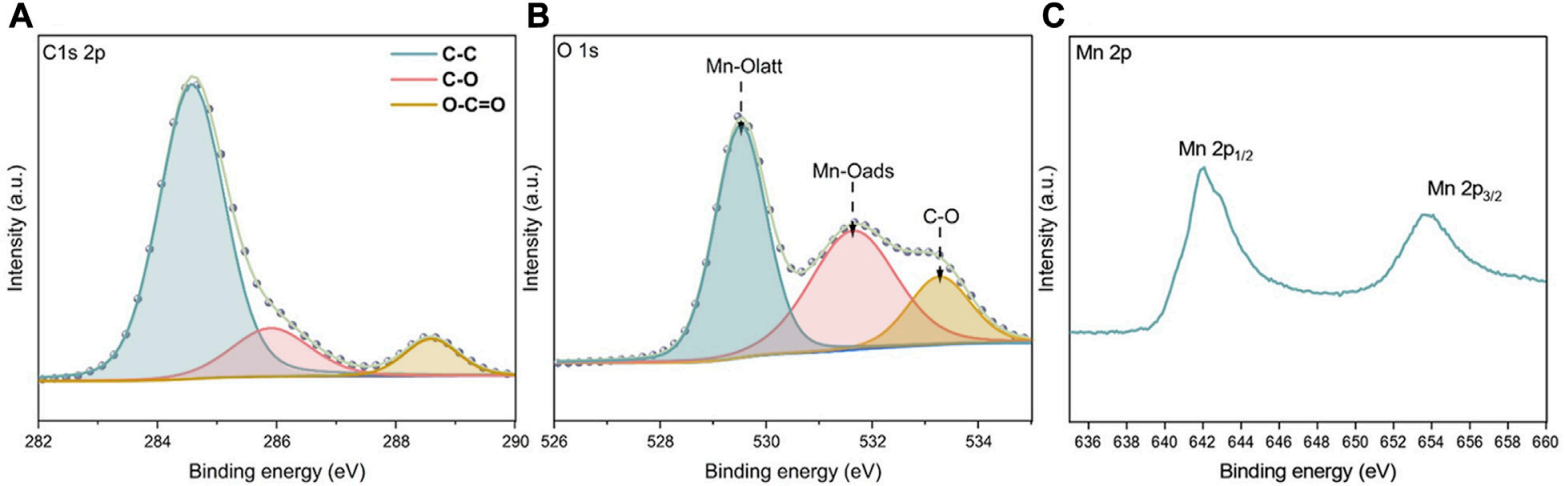
XPS spectra of **(A)** C 1s **(B)** O 1s and **(C)** Mn 2p in 5%G-MnOx sample.

As shown in Figure 6, the sample surface elements were analysed with XPS peak-differentiating and imitating The C 1s spectrum exhibited three peaks, mainly from graphene, with a 284.6 eV binding energy corresponding to C-C moieties, that at 285.9 eV corresponding to C-O moieties and that at 288.6 eV corresponding to O-C=C-O groups, which indicated successful loading of graphene onto the MnO_2_ surface. The O 1s XPS data are also shown in the figure, and the peak at 529.5 eV was generated by the lattice oxygens in MnO_2_ (Mn-Olatt), that at 531.6 eV indicated adsorbed oxygen (Mn-Oads) on the sample surface, while the 533.2 eV peak indicated O-H, C-OH, O=C, *etc.*, Groups on the graphene itself. The Mn 2p spectrum is also shown in the figure, and the two asymmetric peaks at 642.1 eV and 653.7 eV corresponded to the Mn 2p_3/2_ and Mn 2p_1/2_ states, respectively.

**FIGURE 7:**
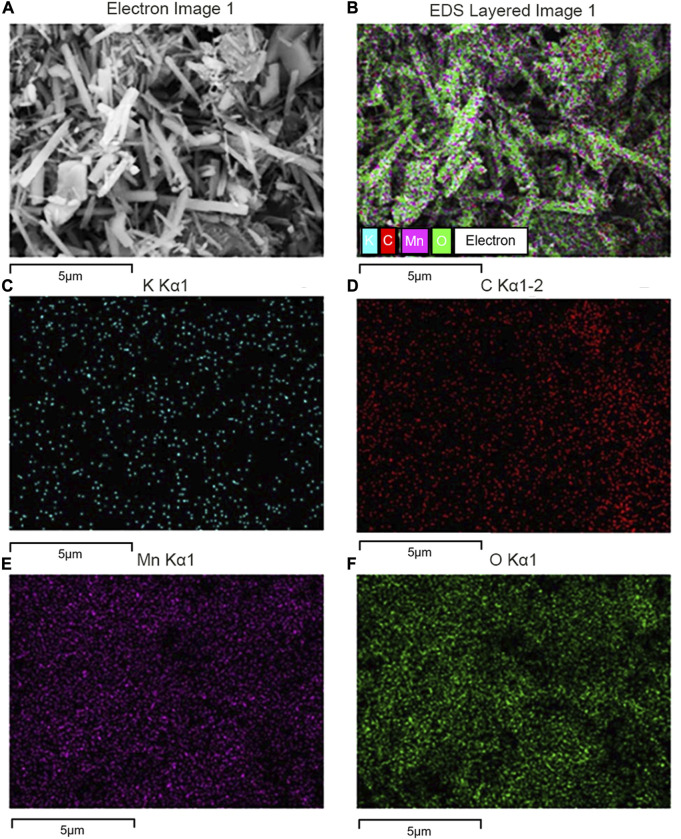
SEM image of G-MnO_2_ sample. **(A)** Electron image 1, **(B)** EDS layered image 1, **(C)** K Kα1, **(D)** C Kα1-2, **(E)** Mn Kα1, and , **(F)** O Kα1.

The SEM image for G-MnO_2-P_ showed in Figure 7 that the material adopted an overall nanorod-like structure, and the surface scan of the EDS energy spectrum showed that C was uniformly distributed on the surface of the sample with a mass fraction of approximately 6.28%, indicating that graphene was successfully doped and grown on the surface of MnO_2_.

**FIGURE 8:**
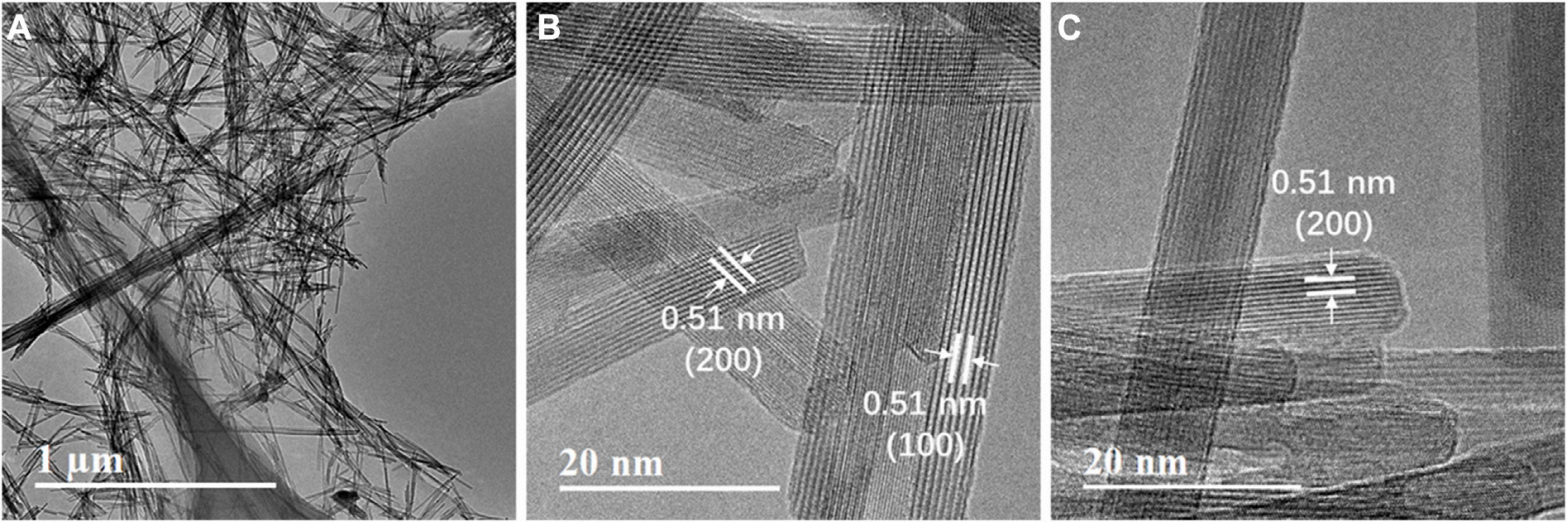
TEM image of G-MnO_2_ sample.

As shown clearly in Figure 8, the 5% G-MnO_2-P_ sample formed morphologically regular nanorods. The HRTEM image showed that the G-MnO_2-P_ nanorods were exposed at the (200) and (110) crystal planes with corresponding crystal plane distances of 0.51 nm and 0.69 nm, which indicated that the 5% G-MnO_2-P_ nanorods were growing in the direction of the (001) plane.

**FIGURE 9:**
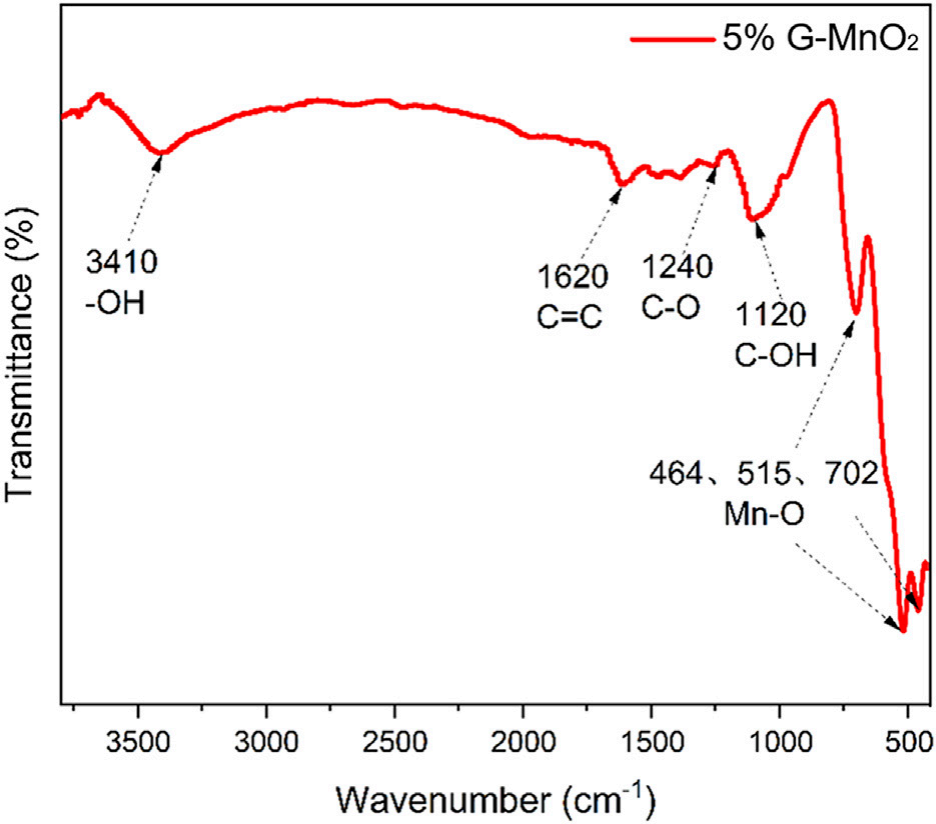
FT-IR spectrum of G-MnO_2_ sample.

The typical peaks for MnO_2_ and graphene appeared in the FT-IR spectrum of G-MnO_2-P_ (Figure 9), which indicated successful preparation of the graphene composites. Of these, the peaks at 464, 515 and 702 cm^-1^ were attributed to the Mn-O stretching vibrations of MnO_6_ octahedra in the 5%G-MnO_2-P_. The peaks at 1,120 and 1,620 cm^-1^ were assigned to C-OH and C=C stretching vibrations, the peak at 1,240 cm^-1^ to a C-O asymmetric stretching vibration and that at 3,410 cm^-1^ to -OH vibrations. The presence of functional groups on the surfaces of the samples increased the affinity of ozone for the catalyst surfaces, which enhanced ozone adsorption on the surface and subsequent dissociation to generate the reactive oxygen radicals involved in the microbial eradication reactions.

### 3.3 Screening of the preparation process parameters

Previous characterizations of graphene-doped samples made via different preparation processes and bactericidal activity experiments revealed that the higher catalytic activities of samples obtained by the one-step precipitation method were mainly due to the higher specific surface areas and surface functional groups. These enhanced the ozonolysis reaction, which generated reactive oxygen radicals in the system and affected the bactericidal performance.

The effects of different graphene doping ratios on the plasma eradication of *E. coli* were investigated. The doping ratios used were 5%, 10% and 20%, and the results are shown in Table 4. The 10% doping level improved the eradication rate to 99.98%, and when the doping ratio was increased further to 20%, the eradication rate did not improve, probably because the excess graphene caused partial coverage of the catalyst surface. The 10% ratio was chosen as the optimum graphene doping ratio for the preparation of high-performance catalysts.

**TABLE 4:** Effect of different doping ratios of graphene on the eradication rate of *Escherichia coli*.

Doping ratios	(%)	10 (%)	20 (%)
Eradication rate	99.93	99.98	99.97

The effect of the plasma action time on the eradication rate for *E. coli* was also investigated. The eradication rates for *E. coli* were 99.82% at 1 min, 99.98% at 2 min and greater than 99.99% at 4 min. With extensions of the action time, the *E. coli* eradication rate increased. It may be that the longer time led to the generation of more active species in the plasma field, which reacted more fully with *E. coli* and provided a much higher bactericidal effect.

### 3.4 Eradication performance tests with different microorganisms

The synergistic plasma and 10% G-MnO_2-P_ method was used to test the eradication rates of three microorganisms, namely, *Staphylococcus aureus*, coronavirus and Aspergillus niger. The results showed that the eradication rate for *S. aureus* was 99.98%, and those for coronavirus and Aspergillus niger were >99.99%, which are remarkable effects. In summary, this method showed excellent effects with a variety of pathogenic microorganisms.

### 3.5 Repeatability experiments

With the 10% G-MnO_2-P_/HAC sample used previously, multiple repeatability cycles were conducted with coronavirus as the target pathogenic microorganism. The eradication rates were greater than 99.99% after 1, 5 and 10 test cycles, indicating that the material had excellent reusability.

### 3.6 Ozonolysis tests

**FIGURE 10:**
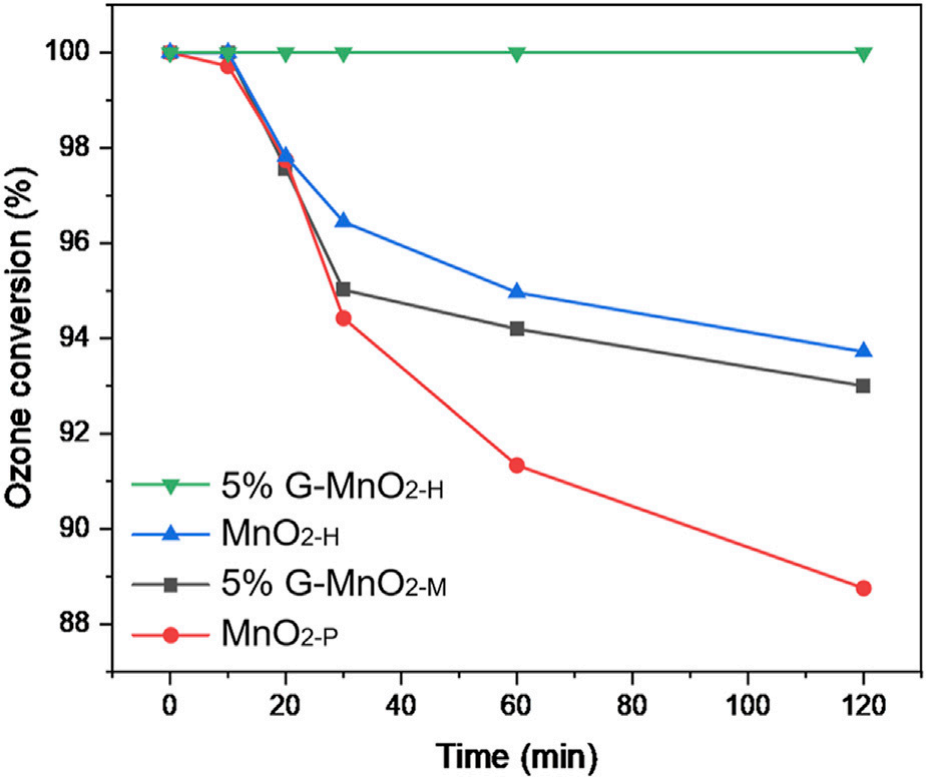
Ozonolysis performance curves for different samples.

The efficiencies for ozone decomposition catalysed by several of the catalytic materials used in this study are shown in Figure 10. The inlet concentration of ozone was 120 ppm, the ambient temperature was 25°C, and the tests lasted for 120 min. Among them, the 5% G-MnO_2-P_ sample showed the best catalytic ozonation performance and maintained 100% activity for 120 min. The activity curve for MnO_2-P_ was close to that for 5% G-MnO_2-M_, and the ozone conversion rate of 94% was maintained for 120 min. The activities of MnO_2-H_ samples were relatively poor, and the ozone conversion rates were 89% after 120 min. The activity for the catalytic decomposition of ozone was comparable with that seen for plasma co-catalysis, and the sequence of the two was consistent, indicating that the efficient decomposition of ozone enabled eradication of the microorganisms.

### 3.7 Mechanistic analysis

**FIGURE 11:**
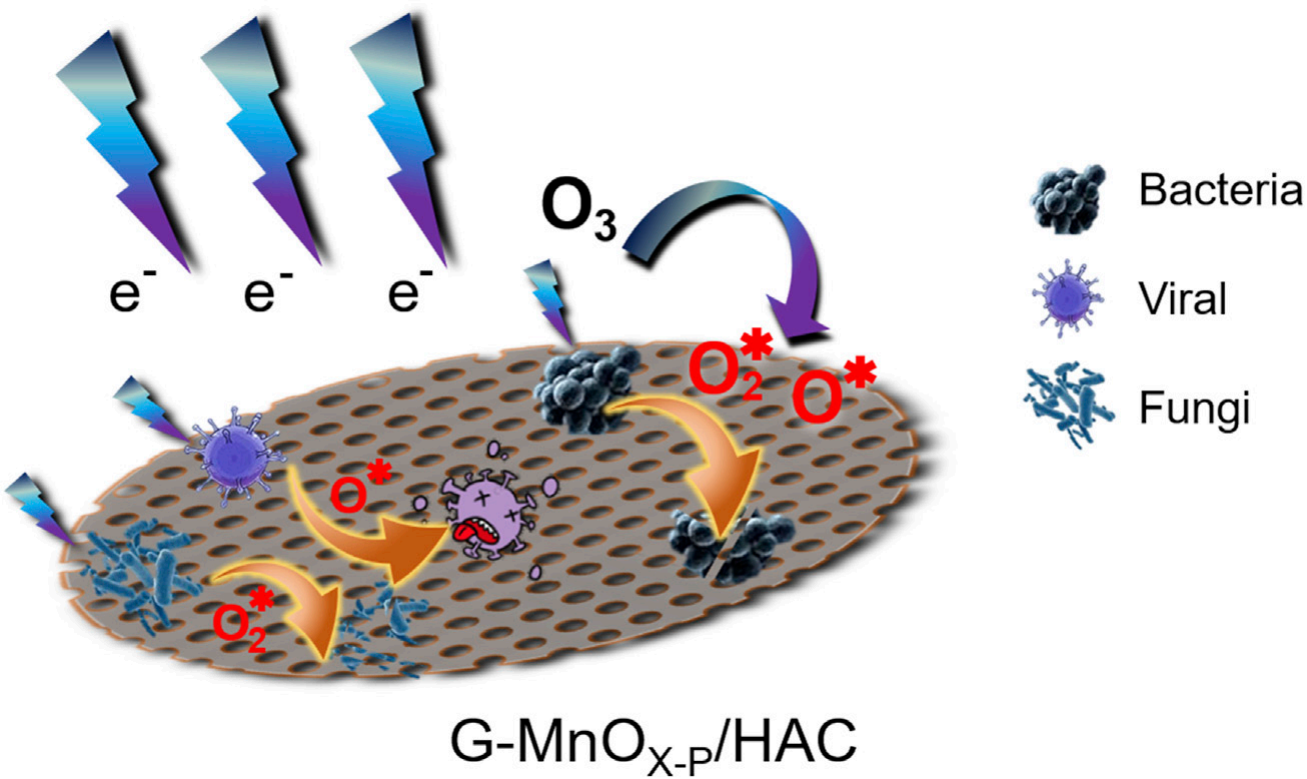
Mechanism of plasma synergistic G-MnO_X-P_/HAC for the eradication of pathogenic microorganisms.

Due to the excellent electrical conductivity, high specific surface area and chemical stability of graphene (Berger et al., 2004; Nair et al., 2008; Kong et al., 2022), it was compounded with manganese oxide to improve the dielectric properties of the material surface and provide efficient generation of the active species. Additionally, the graphene surface groups also enhanced the performance of manganese oxide in the ozone catalytic decomposition process, thus forming more ROS and accelerating the oxidation reaction. Based on characterization of the samples, and the performance of each sample in the synergistic plasma catalytic *eradication* of pathogenic microorganisms, a proposed mechanism is shown in Figure 11. In the plasma system used to generate ozone, the ozone was subsequently decomposed on the surface of the catalyst to generate ROS with stronger oxidation capacities, and the pathogenic microorganisms were eradicated by further oxidation reactions that destroy the cytomembranes and cellular contents. (Yin et al., 2018; Li et al., 2021).

## 4 Conclusion

In this study, the G-MnO_X-P_/HAC sample prepared with a one-step precipitation method and 10% graphene doping had the best bactericidal effect in a DBD reactor after 4 min of action. The eradication rates for *E. coli*, *S. aureus*, coronavirus and Aspergillus niger were all greater than 99.9%. Characterization techniques such as TEM, SEM, XRD, BET and FT-IR showed that the G-MnO_2_ prepared by the one-step precipitation method had a larger specific surface area with more oxygen vacancies and functional groups on the surface, which enabled adsorption of the ozone generated by the dissociated plasma, and ROS formation led to microbial eradication. The performance of the G-MnO_X-P_/HAC was then tested with different microorganisms, and reproducibility experiments were conducted. Finally, the mechanism was analysed with ozonolysis reactions to develop a technology useful for synergistic G-MnO_X-P_/HAC eradication of pathogenic microorganisms in a plasma field.

Microbial air pollution is closely related to human health. The wide range of existing air purification and disinfection technologies and equipment cannot achieve efficient eradication of pathogenic microorganisms through filtration or adsorption alone, which poses challenges for air purification systems designed to eradicate pathogenic microorganisms. This study of G-MnO_X-P_/HAC and the synergistic plasma technology used for pathogenic microbial disinfection provides new research ideas for microbial removal from confined spaces.

## Data Availability

The raw data supporting the conclusion of this article will be made available by the authors, without undue reservation.
